# Can she make it? Transportation barriers to accessing maternal and child health care services in rural Ghana

**DOI:** 10.1186/s12913-015-1005-y

**Published:** 2015-08-20

**Authors:** Kilian Nasung Atuoye, Jenna Dixon, Andrea Rishworth, Sylvester Zackaria Galaa, Sheila A. Boamah, Isaac Luginaah

**Affiliations:** Department of Geography, University of Western Ontario, 1151 Richmond Street, London, ON N6A 5C2 Canada; School of Public Health and Health Systems, University of Waterloo, 200 University Avenue West, Waterloo, ON N2L 3G1 Canada; Department of Geography, University of Waterloo, 200 University Avenue West, Waterloo, ON N2L 3G1 Canada; Department of Integrated Development Studies, University for Development Studies, Wa Campus, Upper West Region, Ghana; Arthur Labatt Family School of Nursing, Health Services Addition, University of Western Ontario, London, ON N6A 5C1 Canada

## Abstract

**Background:**

The Ghana Community based Health Planning and Services (CHPS) strategy targets to bring health services to the doorsteps of clients in a manner that improves maternal and child health outcomes. In this strategy, referral is an important component but it is threatened in a rural context where transportation service is a problem. Few studies have examined perceptions of rural dwellers on transportation challenges in accessing maternal health care services within CHPS.

**Methods:**

Using the political ecology of health framework, this paper investigates transportation barriers in health access in a rural context based on perceived cause, coping mechanisms and strategies for a sustainable transportation system. Eight (8) focus group discussions involving males (*n* = 40) and females (*n* = 45) in rural communities in a CHPS zone in the Upper West Region of Ghana were conducted between September and December 2013.

**Results:**

Lack of vehicular transport is suppressing the potential positive impact of CHPS on maternal and child health. Consistent neglect of road infrastructural development and endemic poverty in the study area makes provision of alternative transport services for health care difficult. As a result, pregnant women use risky methods such as bicycle/tricycle/motorbikes to access obstetric health care services, and some turn to traditional medicines and traditional birth attendants for maternal health care services.

**Conclusion:**

These findings underscore the need for policy to address rural transport problems in order to improve maternal health. Community based transport strategy with CHPS is proposed to improve adherence to referral and access to emergency obstetric services.

## Background

Transport is a critical area in health care access, serving as a link between home and health facilities. In developing countries, poor road network and absence of regular means of suitable transport leaves rural areas inaccessible, making physical access to specialized health care, which is not provided in local health facilities, difficult. This significantly and adversely affects the achievement of maternal and child health outcomes despite tremendous work going on in developing countries [1–5].

Statistics on maternal deaths in Sub-Saharan African countries are alarming. Most of the 40 countries with high levels of maternal mortality are in Sub-Saharan Africa (SSA) and women in the continent face 15 times the risk of dying from pregnancy and childbirth situations compared to women in developed countries [6]. In Ghana, Maternal Mortality Ratio (MMR) reduced by 2.6 % from 1990 and 2010. The period after 2000 witnessed an accelerated reduction of 4.5 %. While indicators are promising, they still leave the country with a MMR of 350 per 100,000 live births [7]. Interestingly, national MMR masks regional disparities, a common characteristic of the health geography of Ghana [8].

Referral, a request for a patient to seek health services at a higher level by a health professional, has been recognized as a critical component in emergency health service delivery. It connects different tiers of health service provision, starting from the lowest level, mostly primary health care units [9]. In maternal health, referral facilitates access to emergency services critically needed to save mothers and children. In an effective and efficient health system, referral can reduce stillbirth by 27 %, neonatal deaths by 18 % and maternal deaths by 50 % [10]. Meanwhile, referral acceptance and compliance is influenced by socio-cultural, economic, geographic, and quality of care at point of service delivery [11, 12].

In promoting referral, distance to facilities has been identified as a critical factor. Apart from its association with delay in reaching the next point, it has the tendency of influencing rejection of referral, which has dire implications for maternal and child health. As a result, studies and interventions have targeted reduction in physical distance to health facilities. However, distance in the context of time spent on reaching the next point as a factor of transportation infrastructure and means of transport have remained understudied [13–15]. This paper examines the challenges of transportation, from a rural community standpoint in Ghana, in relation to physical assess to maternal health care services. In doing so, this paper seeks to highlight the extent to which absence of transportation in a primary health care initiative affects access to maternal health services, and explore strategies for sustainable transportation services in accessing maternal health care in rural Ghana. In addition to this introductory section, the paper is organized into five main sections. The next section discusses the evolution of the CHPS strategy, which is followed by the theoretical context of the study. The methods, comprising the study context and empirical material, then follow. Subsequently, the results, discussion and conclusion are presented.

### Community Based Health Planning and Services (CHPS) strategy

Ghana Health Service adopted the CHPS strategy in 1999 to bring quality and equitable Primary Health Care (PHC) service to the doorsteps of all Ghanaians; particularly, those in rural and hard-to-reach areas. The strategy emphasizes a need to promote the health of communities particularly women and children within the general context of responding to the implementation of MDGs 4 and 5, and the overall developmental goals of Ghana. It presents a shift from institutional and outreach delivery of health to mobile health delivery by placing a resident Community Health Officer (CHO) and a nurse in a community to follow clients to their communities and homes to deliver services. CHPS is underpinned by community mobilization and participation to ensure community support and ownership of the processes. Structures such as the Community Health Committees (CHCs) work in collaboration with the Sub-District and District health management teams to provide leadership for implementation of CHPS. Community Health Volunteers conduct health surveillance and support CHO in health promotion and education in the community. CHOs are provided with basic health equipment and medicines to deliver PHC services and initiate referral to next level health facility for more specialized care during emergencies [16–18].

Indeed, the CHPS zone, represented by a compound, forms the basic unit in the structure of health delivery in Ghana. Although the concept is comprehensively thought out, what seems to be missing in the implementation of this strategy is an understanding of the multi-layered nature of providing adequate health care in remote rural locations. Yet, since the implementation of this policy, there has been no study that focuses on transportation as a key determinant of health care provision in rural Ghana. We use the political ecology of health to understand the effect of transportation barrier to health care services in an environment of multiple restricting factors to health care services.

### The political ecology of health

The study used the Political Ecology of Health (PEH) theoretical framework to examine interactions of sociocultural, political, ecological and economic forces and how their interactions influence access to maternal health in rural Ghana. The phrase ‘political ecology’ when it first appeared in the writing of Blaikie and Brookfield [19], combined ecological concerns with political economy (used in the broad sense of power relations) to study land degradation. Political Ecology (PE) has great strength in combining cultural ecology and political economy into a single analysis framework that has the potential of producing a more comprehensive and holistic analysis of relationships of causal factors at different levels of analysis [20]. In addition, PEH is a strong framework in providing historical narratives of cultural and social factors in the analysis of health. It recognizes that causal factors in health are history laden.

Based on the strengths of PEH, Kalipeni and Oppong [21] used it to study the diffusion of disease in SSA. Also, Richmond, Elliott, Matthews, and Elliot [22] studied health and environment of native populations in Canada using the PEH, while Mkandawire, Richmond, Dixon, Luginaah, and Tobias [23] applied it to examine the spread of Hepatitis B virus in the Upper West Region of Ghana. We conceptualize that transportation for health care is influenced by national and international health policy environment interacting with contextual and compositional factors.

## Methods

### Study context

Ghana’s Upper West Region (UWR) is one of the poorest and least developed regions in the country. In the three regions in the north of Ghana, including the UWR, poverty is endemic. The UWR is the most affected, with 9 out of every 10 persons living on less than US $1.25 a day [24]. The widespread deprivation in the region together with less than desirable roads further challenges community health development. The UWR has a total population of 702,110, with 51.4 % female [25] and only 17.5 % of the total population characterized as urban, compared to national average of 51 % [25]. Endemic levels of poverty contribute to literacy rates much lower than the national average, especially for women [24]. The region has six hospitals and over 53,000 individuals to a doctor in 2013 [8], which is the worse in the country. Only 45.3 % of women in the UWR deliver in a health facility (the second lowest in the country) compared to well over 83 % in the Greater Accra Region, the national capital [24].

This study is concretized on perceptions of community members and community health workers. Although perceptions are subjective, they are shaped by experiences, social-cultural orientations and local environmental factors, thus remain relevant in providing an understanding to a local phenomenon and propounding strategies to fit local context [26, 27]. In addition, data gathering in a perception driven research requires a design that is flexible, non-sequential and allows the research to reshape while in progress, and qualitative research design allows just that [28, 29]. Focus Group Discussions were used to collect data between September and December 2013 in Dornye CHPS Zone in the Wa West District of the UWR of Ghana.

### Focus Group Discussions (FGDs)

FGDs were used, for the main reason that they have strength over other qualitative data collection methods (namely, individual interviews) in gathering perceptions and opinions of several respondents simultaneously and systematically [30] in informal and unstructured setting, which promotes participation and interaction [28, 31]. Further, focus groups were purposively drawn to create separate harmonious groups of males and females to enhance participation of group members and also ensure that gender undertones were captured [32]. Ultimately, FGDs are grounds for peer learning as debates and intense interaction on varied individual perceptions and opinions settle down to consensus group opinions and perceptions, serving as a form of education in several respects and truly becoming the prototype of society [33].

### Sample selection

To ensure perspectives and opinions were diverse, and thematic saturation would be met, the study recruited males (*n* = 40) and females (*n* = 45) of ages between 18 and 70. This age category of participants would have had experiences and perspectives about the delivery of primary health care without a transport strategy. The large age range provided varied experiences on the subject. Community leaders and the CHO were briefed about the study and they subsequently informed particular community members based on their understanding that those were people who could adequately respond to the study questions. As participants arrived, the CHO gave identity numbers in the order of arrival, starting with the first to arrive. The first eleven (11) participants for each of the sexes formed the first group and the others formed the second group for discussion. Table 1 shows the characteristics of the study participants.

**Table 1:** Characteristics of study participants

Characteristic	Male	Female	Total
Sample Size	40	45	85
Marital Status			
Married	27	40	67
Divorced/Widowed/Never Married	13	5	18
Mean age	49.1	40.2	44.5
Economic Activities			
Farming	39	37	76
Trading	0	6	6
Handworks	1	2	3
Mean annual household income in Ghana cedis (GH¢)	369.4	252.2	307.3
Number of Children			
Less than 3 children in household	6	12	18
Between 3 to 5 children in household	20	26	46
More than 5 children in a household	14	7	21
Role in CHPS			
Community Health Volunteers (CHVs)	2	4	6
Community Health Committee (CHC) members	5	4	9
Traditional Birth Attendants (TBAs)	---	4	4
Others	33	33	66

### Data collection

Data was collected using eight focus group discussions, four in each of the two study communities. All group discussions took place outside, in an open, under a tree in both communities. This made the setting familiar, unofficial and unstructured, a precept for participation. An experienced researcher in the team led discussions in Brefo and Waale/Dagaare, the main languages in the study area [28]. A checklist of questions mainly dealing with the relevance of transport in responding to referral or emergency cases, the effects of the absence of transport in the CHPS zone, and strategies for addressing transport issues in CHPS was used. Over all, the checklist only served as a guide, hence allowed related questions to be included and discussed. Discussions were lively, interactive and participatory, and lasted for 1 hour.

### Analysis

With oral consent of participants, all FGDs were recorded verbatim (in Brefo and Dagaare/Waale) and later translated into English by a professional translator and transcribed. Transcribed scripts were read for emerging themes within the context of the study objectives. Reading and re-reading of transcribed scripts brought out categories and their related concepts. Codes were created from concepts and later compiled into a code scheme [34]. Text Analysis Markup System (TAMS) was used in organizing transcribed scripts, documenting categories, coding, and searching results. All transcribed scripts and audio recordings were uploaded onto TAMS for coding and processing. Line-by-line coding and re-coding was employed [35], and to ensure that similarities and differences across the eight focus group discussions were captured, the same coding scheme was applied to all transcripts. In effect, inductive and deductive coding was done [36, 37]. Notable emerging themes included “relevance of transport in CHPS”, “effects of absence of transport”, “coping strategies”, “strategies for sustainable transport” [36].

In ensuring consistency and credibility of data and the findings, considering the fact that qualitative research is largely interpretative and subjective [37], a number of strategies were used. Member checking was one of them. Transcribed scripts and initial emerging themes were sent back to participants and they confirmed two things; first, that the translation from local dialects into English and subsequent transcription were true representation of their discussions, and second that the initial meaning drawn from transcripts was the meaning to their discussions. The second strategy was source triangulation. Checks on transcribed scripts of the eight focus group discussion revealed that the emerging themes reached saturation. Generally, the same themes ran through the different discussion groups. Investigators triangulation was the third strategy used. Two different investigators coded the same portion of a transcribed script using TAMS and concluded on themes and concepts independently. They compared results, discussed and built consensus on the coding process [37].

In order to capture gender-influenced perspectives of the role of transport in delivery of primary health care, attention was paid to gender in the analysis of data. We were also very particular about the differences and similarities of perspectives on the strategies for sustainable transport in primary health from men and women, mainly due to its importance in policy planning and implementation.

#### Ethical approval

The ethical review committee of the University of Western, Ontario, Canada and the University of Development Studies, Wa Campus, Upper West Region, Ghana approved ethics for the research as part of a bigger maternal health research project in the Upper West Region of Ghana. The ethics guaranteed anonymity and confidentially of respondents and responses. It allowed for voluntary participation (entry and exit from the research) at any stage of the research process. Ethical consideration was communicated to research participants in a consent letter, which was endorsed before the start of the research.

## Results

Results of the study are presented under the themes that emerged from the focus group discussions. These are: perceived factors accounting for absence of transport for primary health, effects of absence of transport, and proposed strategies for sustainable transport for primary health care. Under each theme, we present sub-themes that further provide a nuanced understanding of issues in line with our study objectives on transport and rural public health care provision. Quotations from participants are followed by sex (M = male, F = female), age and role in CHPS (V = Community Health Volunteer; C = Community Health Committee member; T = Traditional Birth Attendants, O = Others) to give a mind’s picture of characteristics of respondents.

### Perceived factors accounting for absence of transport

Overall, the respondents indicated that effective transport services serve as the bridge between rural communities and health facilities. In their view, moving patients from their homes to health facilities and subsequently to referral points for specialized care is very important in “village health care”. However, geographic, economic and failure of rural health policies, programs and strategies to adequately address transport in their design were factors accounting for absence of transport for health in rural areas [13].

#### Rural geographies of UWR: hard to reach communities

In relation to what participants referred to as hard to reach communities, poor road network was identified as one of the main characteristics of rural geography of Ghana and this is even more pronounced in the Northern regions. Participants describe the situation with transportation in the area.

“From here to the compound, an individual can only go through a footpath. A car cannot use the road. There is also a big valley on the way and it is not passable during the raining season, from June to October. Those on foot and bicycle swim through but those on motorbike cannot pass (M, 78, C)”.

Women in a focus group explained that other local economic activities such as shea nuts and shea butter businesses are on a small scale; thus, do not attract commercial buyers, which could have led to development of the road network and improvement in transport. Meanwhile, respondents indicated that absence of social services such as educational facilities is another contributory factor. Areas that have expanded educational facilities open up eventually.

#### Poverty and transportation effects

In development literature, road networks and transportation are identified as determinants of poverty. It becomes a dire situation when rural dwellers with low incomes are challenged with availability and high cost of transport when seeking health care.

“We barely can feed ourselves…Even sometimes the transport cost during emergencies is too high and we are unable to afford [it]. When you ask the vehicle owners why they charge such fares, they tell you the roads are bad so it is not their fault” (M, 47, V).

#### Lack of emergency services and transport planning in CHPS strategy

It became clear in the discussions that respondents attribute the absence of transport for primary health delivery to the failure of the CHPS strategy to adequately address the real challenges of healthcare provision in remote places where ambulatory services are not available. Participants suggested that the CHPS strategy ought to have taken into consideration social-cultural, economic, as well as geographic characteristics of rural areas. They attributed the continuous absence of transport for health particularly for referral to absence of transportation in the CHPS strategy. According to them, their expectation for CHPS was to open up the rural areas with motorable roads to facilitate movement of emergency cases to CHPS compound and to the next referral facility. Absence of this “…is the main reason why we still do have women dying during labour…”(M, 67, V). Respondents were quick to acknowledge the use of ambulance in some rare occasions. Even in such occasions participant bemoaned the length of time they had to wait for an ambulance to come from Wa. A participated indicated that she had to wait for more than three hours for an ambulance to arrive from Wa, 40 km away, to convey a woman in labour, because of the difficulties involved in arranging for ambulance and the bad nature of the road.

#### District assembly cannot help: limited resources resulting in low infrastructure

Based on the decentralization system of Ghana, local government as in District, Municipal and Metropolitan Assemblies (MMDAs) are mandated to plan, mobilize resources and carry out developmental projects in their jurisdictions (see ACT 462, Parliament of Ghana, 1993). Besides statutory ceded funding such as the District Assembly Common Fund (DACF) that comes from central government, MMDAs are entreated to mobilize resources to embark on developmental projects. Rural and poor Districts are unable to mobilize the needed resources for road infrastructure, which normally require substantial investment. This was the case in the study area as explained by the District Assemblyperson (the elected representative to the District Assemble):

“Because we are poor, the assembly is not able to generate enough money through levies and taxes to do all these things. It is only recently that few kilometers of roads in the District capital are tarred…”(M, 57, C).

### Perceived effects of lack of transport on health delivery

#### CHPS is unable to leverage health impact

Women who have benefitted from free maternal health care, which is running alongside CHPS in rural areas, spoke of the benefits it brings into their lives. However, they added that the absence of transport is harming its potential impact:

"These days, it is a joy to deliver in a hospital. You don’t pay anything …, it’s absolutely free. This has motivated many of us to visit the hospital for delivery. But the problem is that there is no transport… Here, the roads are so bad. You wonder whether government cares about us. Some of us are forced to deliver at home and that brings complications" (F, 46, O).

Moreover, participants indicated that mortality has not reduced significantly in their community because of absence of transportation to support movement of sick persons to health facilities. This is amply demonstrated in the following statement:

“When they started CHPS here, we were told that dying carelessly, without any serious cause would stop. But still, we continue to see, young women falling sick and dying at home because of difficulty in moving them to the clinic” (M, 60, O).

#### Most Referrals are not honoured because of transport challenges

Referral is a critical component of CHPS. Patients who need specialized care, particularly women in labour, are quickly prepared and passed on to higher point of service. In spite of the critical nature of referral, health workers at the community level are unable to enforce referral due to the financial implication associated with transport and related matters [9]. The nurse is obligated to refer but the decision to adhere to referral lies with the family members of the patient. It becomes difficult to adhere to referral when family members do not have enough money to support it. A health volunteer in a focus group discussion narrates the tragic story of a young woman who passed away at home because the family could not afford the cost of moving her to Wa, which is only 40 km away.

"We all know [name of deceased], we all know how she died. The nurse asked the family to send her to Wa for her to deliver there realizing the complications with the pregnancy. But because the family could not afford the cost, she had to remain at home to deliver and we lost her through that" (M, 45, V).

Respondents indicated that more than 70 % of the cases referred to the hospitals are not honoured mainly due to high cost and unavailability of regular transportation.

It was obvious that some participants did not understand the role of the CHPS strategy. Even though there was community sensitization to explain the CHPS strategy to community members, some community members did not anticipate high rate of referral. Their expectation was that CHPS compounds would act as clinics, “… give us medicine to all our sicknesses” (M, 47, O).

Participants complained of frequent referrals to hospitals in urban centers for care when they struggle to get a patient to the CHPS compound. Coupled with the high cost involved in moving patients to next level and the absence of decent transportation for such service, “people are not enthused about going to the clinic again with their sickness” (F, 27, O).

### Strategies aimed at minimizing transportation challenges

Participants indicated that even though there are serious challenges with transportation in the area, they have had to rely on various desperate strategies to get patients to health facilities in critical situations. These measures include travelling by bicycle, carrying a patient on foot or waiting for the market day truck.

#### Transiting with a bicycle

Participants explained that transporting a patient on a bicycle is the most common means (excluding women in labour) of transporting people to the CHPS compound and beyond. A young man described the process in a focus group discussion:

"We tie a flat piece of wood to the carrier of a bicycle such that the patient can have a good balance sitting at the back. The patient is seated on the wood and tied to the rider with a piece of cloth" (M, 33, O).

Participants indicated it takes about two hours to cover a distance of about 20 Km from the community to the sub-district clinic.

#### On foot, patient on back or being carried by two or more people

Walking for more than 10 km to a CHPS compound to get care is very common in the CHPS zones because in most cases there are no good road networks, only footpaths. Hence, women have to walk to the compound to access antenatal, family planning services, and other health services. It was revealed that in the pre-CHPS period, Traditional Birth Attendants (TBAs) were supporting women through pregnancy and delivery them. With the coming of CHPS, women are encouraged to attend antenatal care while TBAs are stopped from operating in the communities. However, women indicated that walking to the CHPS compound for regular antenatal care was difficult considering the far distance they have to travel when pregnant. 

During emergencies, patients are carried on head to the CHPS compound.

“These days, we rely on a motor tricycle in the community. Even with that people still carry patients during the raining season when the stream cuts off the road. This is how we are managing since there is no regular transport” (M, 45, V).

#### Waiting for the ‘market day truck’

Apart from pregnancy related complication, which is a matter of life and death, other emergency cases are not given rapid response. Instead, local medicine is relied upon to manage illness until a market day when there would be a market car passing through these remote communities. In the study area, a market day is once every six days, implying that a patient may wait for 5 days before having transportation service to access health care. Awareness and education provided under CHPS have engendered confidence of rural communities in orthodox medicines and assured them of access to orthodox health care services.

“Even though we know that a patient should seek medical care immediately, lack of transport would not allow us. So we go back to our fathers’ medicines to make sure the patient is kept alive while we wait for the market day to move to the CHPS compound…” (M, 60, C).

#### Back to indigenous medicines

There seemed to be a growing interest among study participants to go back to traditional medicines as a result of difficulty in accessing orthodox health services. However, males and females had different positions on the use of traditional medicines. From males FGD: “I am convinced that some of them [patients] get cured before they reach the clinic… So for me, there is no need seeking care from the clinic” (M, 57, C). Women rather think otherwise:

"We have all seen the difference between giving birth at home …, and giving birth in a clinic… At least we are more assured of live birth in a clinic. Therefore, taking local medicine is solely a coping measure, while waiting for transport" (F, 36, O).

### Proposed strategies

Social capital is high in the study area and participants indicated that they could make regular contributions towards the cost of transportation and other cost related to referrals.

"We are already helping each other. We can form groups and contribute to the cost of transport when one is sick" (F, 41, C).

Women in the study area mobilize themselves into mother-to-mother support groups to jointly undertake activities and contribute money to support their petty businesses. Young men in the community also form groups to support each other on the form. Participants explained that they could contribute in groups towards cost of accessing health care. On security regular transport, participants suggested that having an agreement with transportation service operators close by was critical. They believe the few transportation operators will be willing to convey emergency cases once payment for the cost of transportation is guaranteed. With the two strategies:

“… a transport plan should be available to help us move our patients from the house to hospital and something small given to patients on referral to help them move” (M, 41, O).

In the long run, community members indicated that they could purchase their own means of transport for emergency transport and advocate for reshaping of their road to allow for all year round use.

“Without roads, government will bring very fine initiatives to improve our health, but they [fine initiatives] will not work. We will remain in this our village and will not have the chance of enjoying them [fine initiatives]. So roads are important for us” (F, 48, V).

**Fig. 1:**
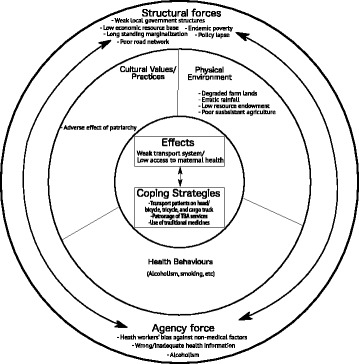
Political ecology of transport effect on maternal health care access in rural Ghana

The findings from this study elucidate the absence of transportation for health in rural Ghana. Using political ecology theoretical framework, we are able to conceptualize interconnections and interrelationships of factors, interacting within a socio-cultural, political and physical environment to create poor transport system for health and low access to maternal health care services in rural communities (see Fig. 1). These factors are cascading and ran through “multiple steps from initial recognition of a problem through access to, and utilization of, specific health services” [2, 38].

**Fig. 2:**
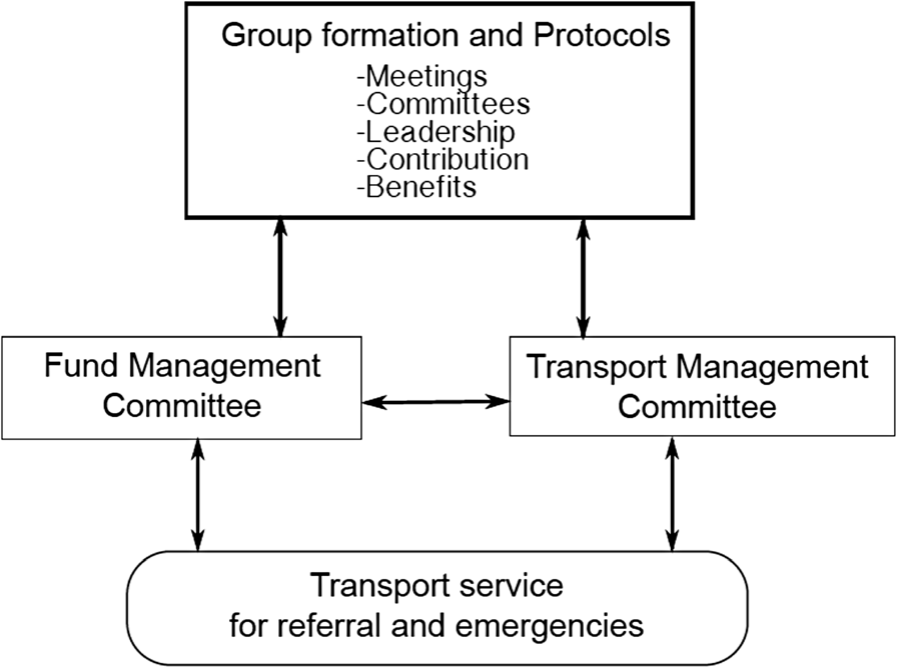
Sustainable community transport system for health access

We categorize these factors into structural and agency forces. Low economic resource base, poor road network, long-standing marginalization, endemic poverty, weak local government structures and policy lapses were identified as structural forces while wrong/inadequate health information and poor prioritization of household expenditure (alcoholism) were the agency forces. These two categories of forces interact to effect and in turn are affected by cultural values and practices, health behaviours, and the physical environment of rural areas, which combine to exacerbate the challenge of health care access. Meanwhile, a consolidation of the proposed strategies provides an opportunity for a community driven sustainable transportation system to support referral and health access for maternal health care services in rural Ghana in particular (see Fig. 2).

## Discussion

Rural areas in developing counties lack transportation for health delivery partly because of the geography of area [4], individual lifestyles, low household prioritization on health, and inadequacy of policy to provide transportation for health delivery as shown by our results. This challenges the effectiveness of primary health delivery programmes and strategies implemented to increase access to maternal and child health services in particular. This paper demonstrates the need for effective transportation in health delivery particularly in developing countries [11, 13] where ambulance services may not be efficient in supporting access to critical health services, and presents a framework for sustainable transportation services driven by community participation.

In the construct of the current paradigm of PHC in Ghana, referral is relied heavily upon because the main services provided under the CHPS strategy are preventive health and basic health care. This makes transportation issues very important in health care access. The finding that transportation is a problem in this rural setting in Ghana challenges the success of the CHPS strategy at increasing access to health services in general, and reducing maternal and child mortality in particular. This finding is consistent with the framework of determinants of skill birth attendants in the delay model by Thaddeus and Maine [13] which highlight transportation as one of the factors influencing delay in reaching health facilities from home and subsequently from first service point to referred facilities.

Moreover, distance has been used as a proxy to health access. A study in Southern Tanzania on distance effect on maternal mortality found that the percentage of live births in facilities decreased with increase in distance to the nearest hospital and health facilities. The study also found that living more than 35 km way from a health facility has high likelihood of experiencing maternal mortality compared to those living within 5 km [11]. A study in Brazil found similar results [39]. Again, following World Health Organization’s guideline on distance to health facility, Ghana Health Service has used 5 km as the standard for establishment of CHPS compounds [16]. In spite of this criterion, the findings in this study indicate a specific need for further consideration of distance in establishment of health facilities. In rural geography of Ghana and other developing countries, accessibility could be greatly affected by the level of development of infrastructure and availability of ambulatory services, hence it will also be important to consider travel time as an important factor in determining the establishment of facilities. After all, delay in this context is influenced more by the nature of the road and availability of transportation and not the physical distance to health facility. This conceptualization of distance is exhibited in Thaddeus and Maine [13]’s three delay concept. An empirical study that has used the delay concept to examine the causes of delay of pregnant women reaching a health facility for emergency obstetric care in Afghanistan found that difficulties in obtaining transport has more than 2.1 times more likely to cause delay compared to those with no difficulty in obtaining transport [40].

However, noncompliance of referral signifies among other things, ineffectiveness of a referral system. In an environment where the health system is effective and referral is supported by working ambulatory system, there is a high likelihood of high referral compliance. The situation in rural Ghana can be attributed to failure of the referral system to connect with the ambulatory system. In Ghana, the ambulance service is organized under the National Ambulance Service, which is detached from the Ghana Health Service, creating challenges in coordination and collaboration in the provision of transportation for referral. Although it may be difficult in organizing transportation service for referral, the results of this study speak to a bigger challenge that goes beyond collaboration gap. It touches the inability of policy to include transportation or ambulatory service in its conceptualization as indicated by study participants. A careful consideration of the geography of rural areas, which the CHPS strategy initial targeted, could have reveal the potential effect of absence of transportation on referral and maternal health outcomes. The relationship between difficulty in finding transportaion and low adherence to referral in developing countries has been well documented in the literature. For instance, a study in Burkona Faso found that during the raining season, complaince in rural was 44% lower [41]. 

Moreover, building a sustainable transportation strategy for health access will require a comprehensive framework that takes onboard structural and agency factors. In respect, it should be able to fit into existing national and local health policies and at the same time take into consideration the geography of rural areas. The suggestion advanced in this paper is a starting point for building this kind of transportation system. We build on a similar system implemented in Northern Nigeria that showed that community level emergency transport systems have the potential of reducing delay in access to emergency obstetric care [2]. Following the findings, we conceptualize a two-component transportation system: resource mobilization to fund transportation cost, and negotiation and management of transport operation (see Fig. 2).

The first stage is mobilization of groups and setting them up with guiding protocols. Protocols may include meeting schedule, leadership terms and responsibilities, membership contributions, terms around membership benefits and formation of committees. The second stage includes setting up of committees. We suggest two committees in our current model but the number can increase depending on group’s decision. The first is a fund management committee, which may perform activities such as opening and managing a bank account, disbursing funds to transport operators for services and also to members referred or transported to health facilities during emergencies. Money disbursed may be treated as a loan depending on the conditions agreed upon by the group. The second committee, a transport management committee will be in charge of negotiating, signing and enforcing the agreements with transport operators. The fund and transport management committees will account to the general membership during meetings and also supervise the quality of service provided to members by transport operators.

Although our model is based on the findings of this study and supported by empirical studies [2], we acknowledge it may require pre-testing and further analysis. In this regard, the CHPS strategy in Ghana, which is centered on community mobilization and empowerment, is a relevant policy space for pre-testing and implementation of the model.

## Conclusion

In conclusion, geographic/physical barriers to health access remains problematic in the context of Ghana’s UWR, where road networks are mostly non-existent. The assumption that a distance of 5 km is a walking distance and as such a facility 5 km away is accessible may need to be further examined, since the study showed that communities are not able to access health facilities due to absence of roads and absence of transport. Ultimately, geographic/physical access needs to be contextualized in terms of travel time and not only in travel distance. Increasing access and compliance to referral will require improvement of road network in rural areas. This study recommends that as a matter of policy, implementation of the CHPS strategy should run concurrently with opening up of rural areas with roads to facilitate health access.

Moreover, emergence of poor economic conditions as a barrier to accessing transport in the study shows that the parameters of transport for health go beyond availability of transport and motorable roads. The sustainable transport for emergency model constructed in this paper could be one of the ways of ensuring availability and affordability of transport for health access. Overall, it is imperative for proactive action on road construction in the Upper West Region, rural Ghana and other developing countries with similar context to improve transportation for rural health with a focus on political ecologies of transport.
